# Supplementary material for: Risk Stratification in Patients with Acute Pulmonary Embolism: Current Evidence and Perspectives
Source: J Clin Med. 2022 Apr 30;11(9):2533. doi: 10.3390/jcm11092533 (PMC9104204; doi:10.3390/jcm11092533)
Supplement: Supplementary file 1 [file jcm-11-02533-s001.zip › jcm-1670117-supplementary.pdf]

Review

Risk Stratification in Patients with Acute Pulmonary Embolism: Current evidence and Perspectives

Antonio Leidi, Stijn Bex, Marc Righini, Amandine Berner, Olivier Grosgurin and Christophe Marti

Supplementary Materials

Supplementary Table S1. Original and simplified pulmonary embolism severity index (PESI and sPESI)

Supplementary Table S2. The Hestia rule

Supplementary Table S3. Components of several prediction scores

Supplementary Table S1. Original and simplified pulmonary embolism severity index (PESI and sPESI).

Variables	PESI	sPESI
**Demographics**		
Age	Age in years	+1 if >80
Male gender	+10	
**Comorbidities**		
Cancer	+30	+1
Cardiac failure	+10	+1 *
Chronic pulmonary disease	+10	
**Clinical signs**		
Pulse ≥ 110 par min.	+20	+1
SBP <100 mm Hg	+30	+1
Respiratory rate ≥ 30 / min.	+20	
Temperature < 36°C	+20	
Confusion	+60	
Oxygen saturation < 90%	+20	+1

* 1 point assigned for Cardiac failure OR chronic pulmonary disease. SBP: systolic blood pressure

PESI Class I <66, Class II: 66 to 85, Class III: 86 to 105, Class IV: 106 to 125, Class V >125 pts

sPESI low risk 0 point.

Supplementary Table S2. The Hestia rule.

**Checklist questions of the Hestia rule**
Is the patient haemodynamically unstable?
Is thrombolysis or embolectomy necessary?
Active bleeding or high risk of bleeding?
More than 24 h of oxygen supply to maintain oxygen saturation >90%?
Is pulmonary embolism diagnosed during anticoagulant treatment?
Severe pain needing intravenous pain medication for more than 24 h?
Medical or social reason for treatment in the hospital for more than 24 h (infection, malignancy, no support system)?
Does the patient have a creatinine clearance of <30 mL/min?
Does the patient have severe liver impairment?
Is the patient pregnant?
Does the patient have a documented history of heparin-induced thrombocytopenia?

Supplementary Table S3. Components of several prediction scores.

Component	Prediction score			
	**ESC**	**Bova**	**TELOS**	**SHIELD**
Age	•			
Gender	•			
Cancer	•			
Cardiac failure	•			
COPD	•			
Chronic renal Insufficiency	•			
Cerebrovascular disease	•			
T° < 36	•			
Shock index				•
Tachypnoea	•			
Confusion	•			
BP < 100 mmHg	•	•		
HR >100-110/min	•	•		
SaO2 < 90%	•			•
Cardiac biomarkers	•	•	•	•
Lactate			•	•
Right ventricular dysfunction	•	•	•	•
